# Between Crigler-Najjar Syndrome Type II and Gilbert Syndrome: Expanding the Spectrum of Uridine Diphosphate Glucuronosyltransferase 1A1 (UGT1A1)-Related Hyperbilirubinemia

**DOI:** 10.7759/cureus.100926

**Published:** 2026-01-06

**Authors:** Carolina Jesus Sá, Rita Vilar Queirós, Tânia Pessoa, Sandra Santos, Susana Castilho

**Affiliations:** 1 Pediatrics, Unidade Local de Saúde (ULS) Arco Ribeirinho, Barreiro, PRT; 2 Pediatrics, Centro Hospitalar Barreiro Montijo, Unidade Local de Saúde (ULS) Arco Ribeirinho, Barreiro, PRT; 3 Pediatrics, Hospital Nossa Senhora do Rosário, Unidade Local de Saúde (ULS) Arco Ribeirinho, Barreiro, PRT

**Keywords:** crigler-najjar syndrome, gilbert syndrome, jaundice, ugt1a1 mutation, unconjugated hyperbilirubinemia

## Abstract

Crigler-Najjar syndrome (CNS) and Gilbert syndrome (GS) are inherited non-hemolytic unconjugated hyperbilirubinemias caused by mutations in the uridine diphosphate glucuronosyltransferase 1A1 (*UGT1A1*) gene, resulting in reduced or absent bilirubin conjugation. These disorders form a phenotypic spectrum, with CNS type I (CNSI) representing the most severe form, CNS type II (CNSII) an intermediate phenotype, and GS the mildest. Differentiation between CNSII and GS can be challenging due to overlapping bilirubin levels and genetic variants.

We report the case of a female newborn of Nepali descent who developed jaundice within the first 24 hours of life, requiring multiple courses of phototherapy. Persistent unconjugated hyperbilirubinemia was documented beyond the neonatal period, in the absence of hemolysis or hepatic dysfunction. Genetic testing identified compound heterozygosity for *UGT1A1 *variants: *c.1456T>G (p.Tyr486Asp)*, associated with CNSII when homozygous, and the promoter polymorphism UGT1A1*28, characteristic of GS. Over time, bilirubin concentrations progressively declined, reaching normal values by 11 months of age, and the patient remained clinically well with normal growth and neurodevelopment.

This case highlights the diagnostic overlap between CNSII and GS, emphasizing the importance of correlating biochemical, genetic, and longitudinal clinical data. The coexistence of pathogenic and polymorphic *UGT1A1* variants likely resulted in an intermediate phenotype, with early CNSII-like bilirubin levels followed by normalization consistent with GS. These findings broaden the understanding of the *UGT1A1*-related hyperbilirubinemia spectrum and reinforce the view that CNS and GS represent a clinical continuum rather than distinct entities. Comprehensive genetic testing and long-term follow-up are essential for accurate diagnosis, prognosis, and family counseling.

## Introduction

Crigler-Najjar syndrome (CNS) and Gilbert syndrome (GS) are inherited non-hemolytic disorders characterized by unconjugated hyperbilirubinemia resulting from mutations in the uridine diphosphate glucuronosyltransferase (UGT) 1A1 (*UGT1A1*) gene. These mutations lead to varying degrees of deficiency in UGT1A1 enzyme activity, the enzyme responsible for the conjugation of bilirubin in the liver, thereby impairing bilirubin clearance. In both syndromes, the clinical phenotype is determined by the serum bilirubin concentration, which reflects the degree of UGT1A1 enzyme impairment and dictates disease severity [1].

CNS is classified into two clinical variants. CNS type I (CNSI) represents the most severe form, characterized by a complete absence of enzymatic activity, resulting in profound jaundice and life-threatening hyperbilirubinemia, with serum bilirubin concentrations ranging from 20 to 50 mg/dL. CNS type II (CNSII) is a milder form of the disease, associated with less than 10% residual enzyme activity and persistent but non-life-threatening unconjugated hyperbilirubinemia (6-20 mg/dL) [2-4]. The estimated prevalence of CNS is approximately 0.6-1 case per million live births worldwide, encompassing both subtypes, with CNSI being the rarer form [5]. In CNS, both sexes are equally affected, with a higher prevalence in children born to consanguineous parents, particularly in CNSI [3].

Gilbert syndrome (GS) is the most common inherited disorder of bilirubin metabolism, affecting approximately 3%-12% of the general population. In GS, the production of the UGT1A1 enzyme is reduced to about 30% of normal levels, and patients experience intermittent jaundice triggered by exacerbating factors. Serum bilirubin concentrations are significantly lower (1-5 mg/dL) than those seen in CNS [2,4,6].

The differential diagnosis of these syndromes is classically based on serum bilirubin concentration and its responsiveness to phenobarbital administration. Phenobarbital induces UGT activity, enhancing bilirubin clearance through increased hepatic uptake, storage, and excretion. In most patients with CNSII, serum bilirubin levels decrease by more than 25% following phenobarbital treatment, as the medication augments residual *UGT1A1* activity, whereas no response is observed in patients with CNSI or GS. For a definitive diagnosis, genetic or molecular testing is necessary to identify pathogenic variants in the *UGT1A1* gene. CNS may result from point mutations, insertions, or deletions within any of the five exons comprising the *UGT1A1* coding region on chromosome 2q37.

GS, on the other hand, usually results from mutations in the promoter region of the same gene, most commonly a polymorphic insertion of additional TA repeats, which reduces transcriptional efficiency [2]. Almost all cases of *UGT1A1* deficiency are transmitted in an autosomal recessive manner, requiring homozygous or compound heterozygous mutations. Because numerous genotype variants produce a range of phenotypes with varying degrees of enzymatic activity and unconjugated hyperbilirubinemia, there is substantial overlap between these disorders. Consequently, these clinical entities are now considered part of a spectrum representing a single disorder with variable severity. Precise differentiation between CNS and GS may therefore not always be possible, particularly in cases of compound heterozygosity involving a coding mutation and a promoter polymorphism [4,6-11].

An inherited non-hemolytic unconjugated hyperbilirubinemia syndrome should be suspected in newborns or children presenting with persistent unconjugated hyperbilirubinemia in the absence of hemolysis or hepatocellular disease. A thorough evaluation, including the assessment of jaundice, the exclusion of alternative causes of hyperbilirubinemia, the serial monitoring of serum bilirubin levels, response to treatment, confirmation through comprehensive molecular genetic testing, and long-term follow-up, is essential to establish a definitive diagnosis. We report a case of a female newborn presenting with persistent neonatal unconjugated hyperbilirubinemia, exhibiting an intermediate phenotype between Crigler-Najjar Syndrome type II and Gilbert syndrome.

## Case presentation

A female newborn of non-consanguineous parents of Nepali descent, with no relevant family history, was born at 37 weeks and six days of gestation at a peripheral hospital.

The pregnancy was well monitored, with negative serologies for HIV, syphilis, hepatitis B/C, and toxoplasmosis, and the mother was immune to rubella, with a blood type of 0Rh-. The prenatal ultrasounds showed no significant abnormalities, and the group B streptococcus screening was negative. The spontaneous rupture of membranes occurred 22 hours before delivery, with clear amniotic fluid. The newborn was delivered via cesarean section (assisted with a vacuum extractor) due to prolonged fetal bradycardia. The Apgar scores were 8/10/10, and the birth weight was 2555 g (26th percentile on the Fenton growth curves).

The newborn developed jaundice within the first 24 hours of life, with a maximum transcutaneous bilirubin level of 12.3 mg/dL, without signs of acholia or dark urine. Examination revealed a caput succedaneum, with no other signs of birth trauma. The baby's blood group was 0Rh+, with a negative direct Coombs test. Intensive phototherapy was initiated. On the third day of life, the serum total bilirubin level was 12.7 mg/dL, with no increase in direct bilirubin. Phototherapy was discontinued; however, the newborn remained hospitalized due to a 9.6% loss of birth weight (2310 g). Formula feeding was introduced as a supplement to breastfeeding to ensure adequate oral hydration. On the fourth day of life, the transcutaneous bilirubin level rose to 16 mg/dL, prompting a second 24-hour course of phototherapy. By day 5, the serum total bilirubin had decreased to 14.1 mg/dL; phototherapy was discontinued, as no further treatment was indicated; and the newborn was discharged home.

At 12 days of age, the newborn was brought to the emergency department due to worsening jaundice, with no changes in feeding, urine or stool output, or vomiting.

Upon examination, the newborn had a weight of 2650 g and marked jaundice involving the entire body, including the palms and soles, with normal heart and breath sounds, a soft abdomen, a normotensive anterior fontanelle, and good muscle tone and reflexes.

Laboratory tests revealed a total bilirubin level of 29.7 mg/dL, with a direct bilirubin level of 0.6 mg/dL. Complete blood count, reticulocyte count, coagulation profile, peripheral blood smear, alanine transaminase (ALT), aspartate transaminase (AST), alkaline phosphatase, gamma-glutamyl transferase (GGT), albumin, urinalysis, abdominal ultrasound, and transfontanellar ultrasound yielded unremarkable results, as shown in Table 1 and Table 2.

**Table 1:** Laboratory results at presentation.

	Patient Value	Reference Range
Hemoglobin	12.4 g/dL	9.4-13.0 g/dL
Leukocytes	10000/L	5000-15000/L
Platelets	383000/L	210000-650000/L
Reticulocytes	1.5%	0.5%-2.0%
Prothrombin time	110%	75%-120%
International normalized ratio	0.9	0.8-1.2
Activated partial thromboplastin time	25 seconds	23-38.4 seconds
Urea	16 mg/dL	10-50 mg/dL
Creatinine	0.20 mg/dL	0.1-0.36 mg/dL
Sodium	137 mmol/L	136-145 mmol/L
Potassium	4.5 mmol/L	3.5-5.1 mmol/L
Chloride	104 mmol/L	98-107 mmol/L
Albumin	3.9 g/dL	3.8-5.4 g/dL
Aspartate transaminase	24 UI/L	<34 UI/L
Alanine transaminase	8 UI/L	<55 UI/L
Gamma-glutamyl transferase	25 UI/L	9-36 UI/L
Alkaline phosphatase	330 UI/L	<500 UI/L
Total bilirubin	29.7 mg/dL	<1.2 mg/dL
Direct bilirubin	0.6 mg/dL	0.1-0.5 mg/dL

**Table 2:** Summary of complementary examination results.

Examination	Results	Description
Peripheral blood smear	Normal	No significant anisocytosis or poikilocytosis observed. No immature or atypical forms. No platelet aggregates
Urinalysis	Normal	Negative for leukocytes, nitrites, proteins, glucose, ketone bodies, urobilinogen, bilirubin, and hemoglobin
Abdominal ultrasound	Normal	Liver with normal morphology and size for age, with regular contours and preserved echotexture. Gallbladder contracted, without intra- or extrahepatic biliary duct dilation. Pancreas and spleen of normal size and appearance, with no structural abnormalities. No free intraperitoneal fluid
Transfontanellar ultrasound	Normal	Linear interhemispheric fissure; lateral ventricles, third and fourth ventricles with normal shape and configuration; symmetrical parenchymal echogenicity without abnormalities. Posterior fossa unremarkable

The newborn was admitted to the neonatology department for further investigation and phototherapy. After completing three days of phototherapy, the total serum bilirubin had decreased to 13.3 mg/dL, below the threshold for continued treatment, and phototherapy was discontinued. Prior to discharge, genetic testing for pathogenic variants in the *UGT1A1* gene was performed, and the patient was referred for a gastroenterology appointment.

**Figure 1:**
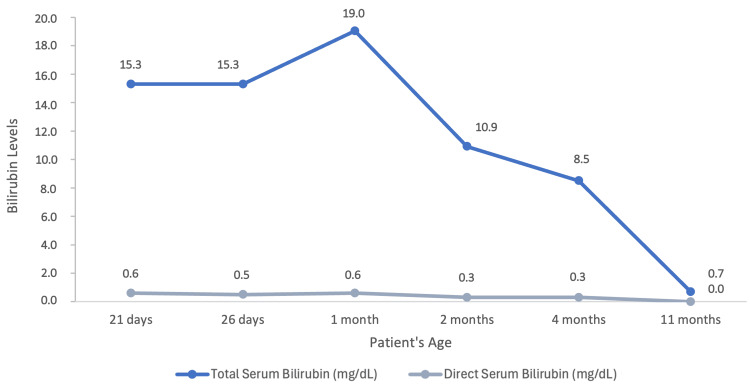
Evolution of total and direct serum bilirubin levels during follow-up according to the patient's age.

In the first two months following discharge, serum bilirubin levels were closely monitored, ranging from 10.9 mg/dL to 19.04 mg/dL (Figure 1), none of which exceeded the age-specific threshold for phototherapy. Since these values seemed consistent with Crigler-Najjar syndrome type II and the baby was asymptomatic with a benign clinical course, it was decided not to perform a phenobarbital challenge test, as CNS type I was considered unlikely. Genetic testing identified pathogenic compound heterozygous point mutations in the coding region of the *UGT1A1* gene on chromosome 2q37 [UGT1A1 (NM_000463.3): c.1456T>G (p.Tyr486Asp)], consistent with Crigler-Najjar syndrome type II, along with a dinucleotide repeat expansion in the TATA box of the promoter region of UGT1A1 [UGT1A1*28 (A(TA)₇TAA promoter polymorphism)], characteristic of Gilbert syndrome. Parental testing for similar mutations was not possible; therefore, the mode of inheritance in this case is unknown.

During follow-up, the patient was closely monitored through regular appointments in general pediatrics and pediatric hepatology, with serial measurements of serum bilirubin levels during the first year of life. As illustrated in Figure 1, her bilirubin levels progressively decreased, reaching normal values by 11 months of age. She was exclusively breastfed until six months of age, at which time complementary feeding was introduced without evidence of food allergies. Breastfeeding was continued until 12 months of age. She maintained normal growth and neurodevelopment, with no reported health complications, and continued follow-up until 12 months of age, at which point she was discharged from hospital care and remained under routine surveillance by her primary care physician.

## Discussion

Inherited non-hemolytic unconjugated hyperbilirubinemias, such as CNS and GS, should be considered in neonates or children with persistent unconjugated hyperbilirubinemia in the absence of hemolysis. More common causes in the neonatal period, including physiologic jaundice, breast milk jaundice, and isoimmune hemolysis, should be excluded first.

CNS and GS can usually be differentiated by the degree and persistence of hyperbilirubinemia. CNSI is the rarest and most severe form, characterized by life-threatening bilirubin levels, whereas GS represents the mildest phenotype. Differentiating CNSII from GS can be challenging, as overlapping bilirubin levels have been reported; however, hyperbilirubinemia in CNSII typically shows greater persistence [4,7].

The present case illustrates these diagnostic challenges. The neonate in question presented with jaundice within the first 24 hours of life, requiring multiple courses of phototherapy. The persistence of unconjugated hyperbilirubinemia beyond the neonatal period, in the absence of hemolysis (normal hemoglobin and reticulocyte count and a negative Coombs test), raised suspicion for an inherited disorder of bilirubin metabolism. Given the absence of signs of liver dysfunction and bilirubin levels ranging from 10.9 to 29 mg/dL, CNS emerged as a primary differential diagnosis.

A phenobarbital challenge test was not performed, as bilirubin levels remained below 20 mg/dL during follow-up, rendering CNS type I unlikely. In the absence of associated symptoms such as pruritus, the clinical team opted to await genetic confirmation while monitoring the patient's clinical status.

*UGT1A1* gene variants underlie both CNS and GS, with disease severity depending on the specific mutations and their impact on enzyme activity. Most cases follow an autosomal recessive inheritance pattern, requiring homozygous or compound heterozygous alleles. In our patient, genetic testing revealed compound heterozygosity for two *UGT1A1* variants: *p.Tyr486Asp*, a missense mutation strongly associated with CNSII when homozygous, and UGT1A1*28, the common promoter polymorphism that accounts for most GS cases in Caucasian and African populations [1,4].

Based on genotype and bilirubin levels, an initial diagnosis of CNSII was assumed. However, bilirubin levels normalized by 11 months of age, favoring a diagnosis of GS. This case therefore represents an intermediate phenotype, with early CNSII-like bilirubin levels, followed by normalization consistent with GS. The coexistence of *UGT1A1* mutations, together with immature neonatal liver function at this age, leading to reduced baseline *UGT1A1* activity, may explain the exaggerated neonatal hyperbilirubinemia, possibly further compounded by breast milk jaundice through the inhibition of bilirubin conjugation.

Although case reports and cohort studies describing phenotypic overlap between CNSII and GS are limited, the identification of patients with diverse *UGT1A1* variants supports the notion that CNSII and GS represent a spectrum rather than distinct clinical entities [6,10]. This case underscores the importance of integrating genetic analysis with longitudinal clinical follow-up, providing valuable insight into the variable expression of *UGT1A1*-related hyperbilirubinemias and informing diagnosis, prognosis, and management.

## Conclusions

This case underscores the complexity of diagnosing and differentiating inherited non-hemolytic unconjugated hyperbilirubinemias in neonates, broadening our understanding of the genotype-phenotype relationship in CNS and GS. Although early bilirubin levels and clinical course suggested CNS type II, genetic analysis combined with longitudinal follow-up revealed an intermediate phenotype with a benign course more closely resembling GS. The recognition of overlapping genotypes and phenotypes in *UGT1A1*-related disorders is essential, as accurate classification has important implications for prognosis, management, and family counseling.
